# Eye–Hand Coordination Impairment in Glaucoma Patients

**DOI:** 10.3390/ijerph16224332

**Published:** 2019-11-07

**Authors:** Teresa Zwierko, Wojciech Jedziniak, Piotr Lesiakowski, Marta Śliwiak, Marta Kirkiewicz, Wojciech Lubiński

**Affiliations:** 1 Institute of Physical Culture Sciences, Laboratory of Kinesiology in Functional and Structural Human Research Center, University of Szczecin, 70-240 Szczecin, Poland; wojciech.jedziniak@usz.edu.pl; 2 Department of Physical Education and Sport, Pomeranian Medical University, 70-123 Szczecin, Poland; lesiakowskipiotr@gmail.com; 3 II Department of Ophthalmology, Pomeranian Medical University, 70-111 Szczecin, Poland; mkierznowska@gmail.com (M.Ś.); martakiszkielis@gmail.com (M.K.); lubinski@pro.onet.pl (W.L.)

**Keywords:** glaucoma, perimetry, eye–hand coordination

## Abstract

This study examined whether patients with glaucoma exhibit differences in eye–hand coordination tasks compared to age-matched normal-sighted control subjects. Twenty-eight patients with moderate-to-advanced stages of glaucoma and 28 subjects with no ocular disease participated in the study. The Motor Performance Series (MLS) of the Vienna Test System including aiming, linear tracking, tremor, and tapping tests were used to assess eye–hand coordination. Monocular Humphrey Visual Field and binocular Humphrey Esterman Visual Field tests were used to estimate visual field (VF) defect severity. Correlation between MLS scores and VF defects, visual acuity, and patient age were assessed. Glaucoma patients performed slower aiming at targets, committed more errors, and took longer to complete linear tracking and tremor tasks compared to the normal-sighted control group. Furthermore, tapping test scores indicated reduced hand movements at maximum frequency. The presence of asymmetrical monocular VF defects were associated with longer error durations in linear tracking tasks. Furthermore, MLS scores decline with advancing age and reduced visual acuity. Glaucoma patients had lower values for most MLS parameters compared to controls. However, monocular and binocular VF defects cannot fully explain the impartments in eye–hand coordination associated with glaucoma.

## 1. Introduction

Glaucoma is clinically defined as progressive damage to the optic nerve and visual field (VF) loss principally associated with a loss of retinal ganglion cells and is widely reported as a leading cause of irreversible visual impairment and blindness [1,2]. Increased intraocular pressure is a major risk factor for glaucoma [3]. The number of people with glaucoma worldwide is anticipated to increase to 80 million by 2020 and 112 million by 2040 [4]. The risk for developing glaucoma increases with age. Prevalence increases from 3.4% at age 73 to 74 years to 9.4% among those 75 years and older [5].

Visual impairment can significantly affect health-related quality of life and increase the risk of injury [6,7]. Limited physical activity due to fear of falling is extremely common among people with eye diseases causing visual impairment. Wang et al. [8] reported that adults with glaucoma are about three times more likely to be inactive in their daily lives than normal-sighted control subjects. Typically, fear of falling is the main reason many glaucoma patients avoid activities such as traveling out of town, walking, or venturing outside their home [8,9]. Furthermore, several studies showed a negative impact of glaucoma on physical and mental health including mobility [9,10], driving ability [11,12,13,14], reading [15], cognitive function [16,17,18], as well as processing speed and other abilities associated with complex yet instrumental activities of daily living [19,20,21].

Eye-hand coordination is required for many basic tasks of daily living such as reaching and grasping objects, meal preparation, writing, using the telephone, eating, and working [22]. To coordinate accurate and/or fast movements, the motor control system must adapt via dynamic changes in the musculoskeletal system and configuration of body segments [23]. For effective eye-hand coordination, several sensorimotor systems, including the visual system, central processing and effector components, must function synergistically. In particular, integral components of eye–hand coordination task performance include projection of the VF onto the retina, sensory transmission of information to the visual cortex, cognitive planning and motor programming, activation of arm muscles to initiate a particular action, focus of attention, and visual feedback [24]. During goal-directed hand movements towards a target, both monocular (accommodation, contrast sensitivity, and perspective) and binocular (retinal disparity and convergence) information are used [25].

Co-ordination of eye and hand movements is affected by ageing. Coast et al. [26] observed that older adults move more slowly than younger adults during a complex reaching task, reaching lower peak velocities and taking longer to complete the movement. Additionally, increased complexity of motor tasks is known to enhance difficulties for older adults in controlling manual movements [27]. Glaucomatous VF defects may worsen problems with eye–hand coordination in older adults. Visual field deficits reportedly cause impairment in initial movement planning and control of eye–hand coordination tasks. Kotecha et al. [28] analyzed the reach-to-grasp behavior of patients with glaucoma compared to normal-sighted control subjects and observed delays in average movement onset (mean delay 100 ms; *p* < 0.0001) and overall movement time (mean 140 ms; *p* < 0.05) in glaucoma patients. Moreover, impairment was correlated with both increasing VF defect severity and impaired stereoacuity. Pardhan et al. [29] studied the reach-to-grasp and transport-to-place tasks used to accurately move an object to a new location and found that patients with glaucoma made more errors compared to the control group. However, no significant differences (*p* > 0.05) were observed between glaucoma patients and the control group in other phases of kinematic movement.

Widespread effects of glaucoma on perceptual and visuomotor processing are unsurprising given that vision provides a key sensory input necessary for behavioral functions requiring controlled, accurate, and rapid movements regardless of whether objects are manipulated directly or using tools [25]. Nevertheless, testing the impact of age-related eye degeneration on functions of everyday life is important for preventing disability and reducing the risk of social isolation. Therefore, the aim of this study was to investigate the influence of moderate to advanced stages of glaucoma on eye–hand coordination. Herein, we attempt to identify the fine motor skills among aiming, linear tracking, tremor, and hand tapping most affected in patients with glaucoma. Patients with glaucoma are hypothesized to have poorer scores in eye–hand coordination tasks relative to those with normal vision.

## 2. Materials and Methods

Fifty-six subjects participated in the study: 28 adults aged 66.82 ± 6.51 years with moderate to advanced stages of glaucomatous optic neuropathy (according to the European Glaucoma Society classification) at least in one eye, and 28 control volunteers aged 65.54 ± 5.12 years with no history of ocular disease. Patients with glaucoma were recruited from the Clinic of Ophthalmology of the Pomeranian Medical University in Szczecin, Poland, and had at least a four-year history of glaucoma treatment. The exclusion criteria were as follows: (1) glaucoma with unstable intraocular pressure; (2) systemic diseases with known effects on retinal function (e.g., diabetes); (3) neurological diseases (dementia, diseases that cause tremor in the extremities, e.g., Parkinson’s disease, patients with a history of brain tumors); (4) severe cardiovascular diseases; and (5) other ocular diseases including cataracts. Demographic and ophthalmological characteristics of participants are presented in Table 1.

The study was conducted in accordance with the Declaration of Helsinki and approved by the local bioethical committee (No. 10/KB/VI/2017). Before examination, subjects were informed about the testing protocol. All subjects signed a written informed consent and were permitted to withdraw from the study at any time.

### 2.1. Clinical Tests

**Figure 1:**
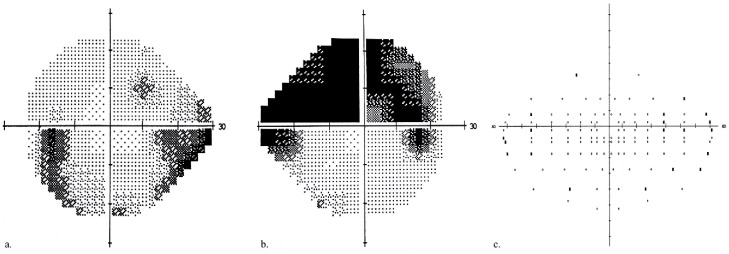
Example of a visual field defect in a glaucoma patient (*gray scale*): (**a**) left eye MD of −4.79 dB, (**b**) right eye MD of −14.64 dB, (**c**) binocular VF defect of 20 (out of 120), and a Esterman coefficient score of 83.

All participants underwent best-corrected visual acuity (BCVA) assessments using a Snellen chart, perimetry (Humphrey Field Analyzer, Carl Zeiss Meditec, Inc., Dublin, CA, USA), and 24-2 Swedish interactive threshold algorithm (SITA)-standard VF testing. Mean deviation (MD) scores were recorded for each eye. A binocular VF score was generated by binocular Humphrey Esterman Visual Field (HEVF). In the HEVF, a grid of 120 test points with light intensity of 10 dB was used to examine more than 130° of the VF. Binocular VF defects with a number of omitted (unseen) points and Esterman coefficients were analyzed. Eyes were classed as “better” or “worse” eye based on the MD score. Inter-eye differences in the MD were calculated and an inter-eye difference of >5 dB was used as the threshold for MD asymmetry. An example of a VF defect is presented in Figure 1.

### 2.2. Measurement of Eye–Hand Coordination

**Figure 2:**
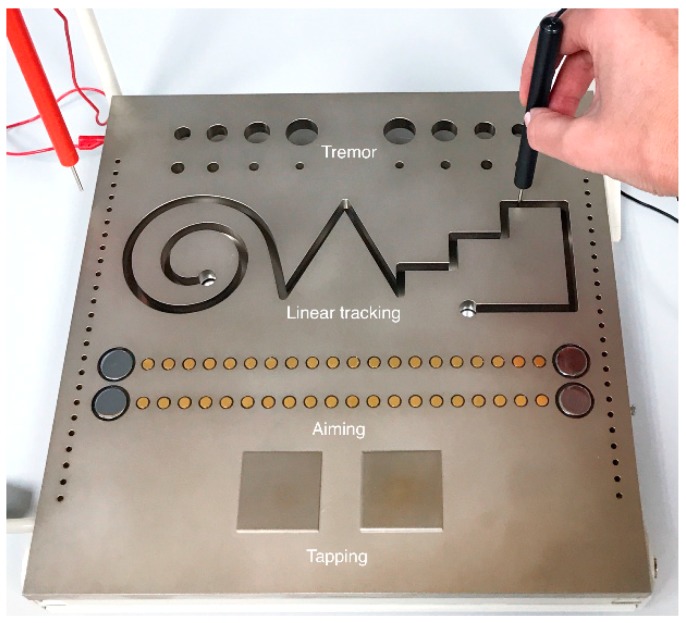
The MLS work panel.

The Motor Performance Series (MLS) of the Vienna Test System (version 8.0; Schuhfried, Austria) was used to assess eye–hand coordination during the performance of both static and dynamic tasks. The MLS consists of a standardized work panel (300 × 300 × 15 mm) with holes and contact fields for various subtests. One of two types of pens was selected (depending on hand preference) for testing perceptual motor skills and the number and duration of contact between the pen and test board were measured as closures in the electrical circuit (5 V, 20 mA). Four eye–hand coordination skills were assessed: aiming, linear tracking, tremor (steadiness), and hand tapping. The MLS work panel used for the eye–hand coordination tasks is presented on Figure 2. All tests were performed with one hand (using the preferred hand) and data were transferred via an interface to a computer system for analysis. The following tests were carried out:

(1) Aiming. The pen was used to touch 20 sensors placed in a row on the board as quickly as possible. The time to perform the test (t) in seconds (s) and number of hits (n) were measured. The aiming index (ai), defined as the ratio of the number of targeted hits to the time taken to perform the test, was calculated, ai = n/t (s^−1^).
(2) Linear tracking. The pen was used to follow a groove with several bends, angles, and curves without touching the sides or bottom of the baseplate. The number of errors (n; i.e., scored by the number of times the pen touches the surface), error duration (s), and time taken to perform the task (t) in seconds (s) were measured. The linear tracking index (ti) was calculated as the number of target errors to the time taken to perform the test, ti = n/t (s^−1^)
(3) Tremor test (steadiness). The task involved keeping the pen inside a hole (inner diameter of 5 mm) on the board without touching the walls for a specified time (32 s). The task was assessed based on the number of errors (n; i.e., scored by the number of times the pen touches the wall) and the average error duration (s).
(4) Tapping test. The pen was used to strike a special 40 × 40 mm square surface as many times as possible within a specified time (32 s). The number of accurate hits (n) was used to determine the wrist–finger speed of untargeted movements with maximal frequency.

### 2.3. Statistical Analysis

Descriptive statistics were performed on all data. The assumption of normality was examined using the Shapiro–Wilk test. Sample data did not follow a normal distribution (*p* < 0.05). For each participant, the median value was calculated for all eye–hand coordination parameters. Values obtained from patient and control groups were compared using a nonparametric Mann–Whitney U-test. For pairwise comparisons, magnitudes of the effect sizes were determined using the *r* value proposed by Cohen [30]. According to Fritz et al. [31], *r* can be calculated as an effect size for the Mann–Whitney U-test using the formula r = z/√N. Cohen’s guidelines for *r* characterize effect size as large (0.5), medium (0.3), and small (0.1). Correlation between eye–hand coordination parameters and VF defect severity and glaucoma patient age were assessed by Spearman’s rank correlation. Values of *p* < 0.05 were considered statistically significant.

## 3. Results

Descriptive statistics of eye–hand coordination parameters and intergroup differences between glaucoma patients and the control group are presented in Table 2.

Analysis of the aiming tests shows statistically significant intergroup differences (*p* < 0.001) for total time and aiming index. Compared to the control group, glaucoma patients required more time to perform aiming tasks (9.801 ± 2.052 vs. 7.982 ± 1.596 s, *p* < 0.001, effect size = 0.46) and as a consequence, obtained lower aiming index values (2.029 ± 0.356 vs. 2.550 ± 0.490, *p* < 0.001, effect size = 0.47). However, the number of hits did not differ between groups (19.214 ± 1.134, *p* = 0.491).

Analysis of the linear tracking test data indicates a higher number of errors for glaucoma patients vs. the control group (31.610 ± 11.100 vs. 24.286 ± 8.059, *p* = 0.020, effect size = 0.31), longer error duration (2.929 ± 1.438 vs. 2.046 ± 0.812 s, *p* = 0.006, effect size = 0.37), and lower tracking index values (0.168 ± 0.099 vs. 0.214 ± 0.091, *p* = 0.035, effect size = 0.28). However, the total time taken to perform linear tracking tasks did not differ significantly among groups (25.574 ± 11.006 for glaucoma patients vs. 25.329 ± 12.651 for the control group, *p* = 0.724).

Tremor test results show that glaucoma patients tended to make more errors (3.964 ± 7.280 vs. 1.107 ± 2.671, *p* = 0.011, effect size = 0.34) with increased error duration (0.219 ± 0.286 vs. 0.066 ± 0.205 s, *p* = 0.014, effect size = 0.33) than the control group with normal vision. Similarly, glaucoma patients also performed poorly in tapping tests compared to the control group (183.036 ± 19.416 vs. 199.929 ± 14.934, *p* = 0.003, effect size = 0.40).

The next stage of statistical analysis focused on investigating whether correlations exist between eye–hand coordination parameters, patient age, and VF defect severity (Table 3). Only glaucoma patient data were included for these analyses.

Significant correlations were found between three eye–hand coordination parameters and age of the glaucoma patient (Table 3). It was confirmed that the aiming index decreases with increasing age (R = −0.374; *p* < 0.05) and the number of errors also increases (R = 0.419; *p* < 0.05), as well as the error duration (R = 0.391; *p* < 0.05) in the tremor task.

In glaucoma patients, lower visual acuity in the better eye is associated with increased time taken to perform the aiming task (R = −0.463; *p* < 0.05) and a lower aiming index (R = 0.507; *p* < 0.05). Lower visual acuity in the better eye is correlated with a greater number of errors (R = −0.444; *p* < 0.05) and increased error duration (R = −0.465; *p* < 0.05) in the tremor test. The linear tracking analysis showed a positive correlation between lower visual acuity in the worse eye and time of error (R = −0.432; *p* < 0.05).

A positive correlation was found between MD scores in the worse eye and error frequency in linear tracking tasks (R = −0.441; *p* < 0.05). This suggests a higher VF defect severity assessment of the worse eye is associated with more frequent errors during tracking tasks. A similar relationship between mean MD asymmetry and error frequency was observed in the linear tracking task (R = 0.405; *p* < 0.05). However, in the other cases, monocular as well as binocular VF defect severity did not appear to affect the eye–hand coordination parameters.

## 4. Discussion

The present study investigated the effects of moderate to advanced stages of glaucoma on eye–hand coordination tasks. The findings showed lower values for most of the analyzed eye–hand coordination parameters (8 out of 10) in glaucoma patients compared to the normal-sighted control group. Statistical significance was supported by medium to large effect sizes (ranging from 0.3 to 0.5). In tasks requiring precise and accurate movements, glaucoma patients performed slower (aiming test) and made more errors of longer duration (linear tracking test) than controls. Moreover, tremor test and tapping test scores show a reduction in steadiness (the ability to adopt and maintain a particular eye-arm/hand position with as little change in position as possible) and maximum frequency movements.

Based on our findings, eye–hand coordination deficits are observed in patients with glaucoma compared to age-matched normal-sighted control, thus, supporting previous observations. For instance, results of a study carried out by Kotecha et al. [28] showed that patients with glaucoma exhibit delays in planning and initiation of reaching-to-objects movement during reaching-and-grasping tasks. Movements were slower and more tentative compared to the control group and differences in grasp-posture programming or grip execution were also observed.

Surprisingly, these results cannot be fully explained by defects in the VF. Only one significant correlation was found between VF defects and eye–hand coordination movements, i.e., MD scores in the worse eye were significantly correlated with the duration of error in the linear tracking task (Table 3). Linear tracking is the most complex task in the MLS suggesting VF defects may be linked to difficulties in performing this type of task. However, studies based on self-reported functional ability to perform daily tasks indicate a weak correlation between task difficulty and VF defect severity in glaucoma patients [32,33]. Pardhan et al. [29] observed that deficiencies in eye–hand coordination measured during reach–grasp–transport object tasks due to glaucoma were more strongly associated with central VF defects owing to age-related macular degeneration than peripheral VF defects. In contrast, Kotecha et al. [28] postulated that VF defects in glaucoma are correlated with longer overall movements, reaching maximum speeds, and earlier deceleration during reaching-and-grasping.

Impaired efficacy of eye–hand movements in our patients is likely to be deepened by the presence of asymmetrical VF defects. We detected significant correlation (*p* < 0.05) between MD asymmetry and duration of error in the linear tracking task. However, in the present study, no correlation between binocular VF defects and eye–hand coordination tasks were observed. In contrast, Lombardi et al. [34] found integrated binocular VF to be significantly correlated with movement onset in glaucoma patients reaching and grasping small objects on a kitchen work surface. In addition, Esterman binocular VF appeared to be significantly correlated with mobility time in obstacle avoidance tasks on an artificial street. In the present study, eye–hand coordination scores were lowered by the presence of asymmetrical VF defects but not influenced by binocular deficits. This observation is similar to the results of Kotecha et al. [28] who noted that planning and online control in reaching-and-grasping movements in patients with glaucoma are impaired by asymmetrical VF defects and to a lesser extent, binocular deficits. Alternatively, it has been suggested that the monocular VF test is more efficient than the binocular VF test in diagnosing VF defects in glaucoma patients [35]. Furthermore, Xu et al. [36] postulated that significant Esterman binocular VF defects only occur when both eyes show severe damage. Therefore, relationships between fine motor skills and VF defects mainly tend to appear in monocular tests, which provide more specific information about the location and depth of defects [35].

Poor efficiency of eye–hand coordination tasks were previously shown to be associated with lower visual acuity [29,37]. Similarly, we found five positive correlations between eye–hand coordination parameters and visual acuity (Table 3). Pardhan et al. [29] concluded that reduced VA due to any pathology affecting central vision results in more corrective movements, increased wrist height, needing more time to make online corrections and reduced overall speed of movements. While central visual acuity is relatively well preserved until the late stages of glaucoma [38], Kim et al. [39] observed a reduction in BCVA in 25% of patients with severe glaucoma. Visual acuity is also important for fine motor skills. Previous studies have observed symptoms of abnormal fixational eye movements in individuals with reduced BCVA [40,41], and reduced BCVA may cause fixation instability. These changes are likely to have a negative impact on visuomotor behaviors, such as linear tracking, aiming and tremor tasks, which was also noted in our study. However further investigations are required to verify these findings.

Significant reductions in most of the coordination parameters in our observations provide some evidence that patients with glaucoma may have longer neural processing times. Glaucoma is a group of progressive optic neuropathies and may lead to impaired neural conductivity [42]. Visual evoked potential (VEP) measurements are used for diagnosing optic nerve dysfunction [43]. Electrophysiological evidence exists in the form of recorded pattern reversal of VEPs in glaucoma patients indicating a deterioration of functional integrity of the visual pathway, shown by an increase in N145 latency and decrease in N75, P100, and N145 amplitudes [44]. Parisi et al. [45] observed a highly significant positive correlation between the P100 amplitude (VEP testing) and VF damage (MD values) in open-angle glaucoma patients. Moreover, they noticed a negative correlation between MD scores and the latency time of the P100 parameter. Electrophysiological impairment may have consequences in terms of functional performance. Geruschat and Turano [46] reported slower reaction times (RTs) during walking in patients with glaucoma compared to the control group. In addition, RT increased with increasing VF defect severity.

Impartments in eye–hand coordination start to appear with advancing age [26,47,48,49]. In our study, a decrease in the aiming index and deterioration in tremor task parameters occurred with increasing age. Aging differentially affects the components of movement kinematics. On the one hand, it has been reported that decline in eye–hand coordination may be due to age-related changes in strategic eye muscle control during task performance. To move the hand to a particular location, the eyes are shifted by rapid eye movements called saccades and foveal fixation is placed on the object of interest. Proper coordination of eye movements is necessary to effectively implement motor tasks. Rand and Stelmach [50] observed compromised eye-movement control in older adults during aiming tasks. Older adults tend to make hypometric primary saccades followed by corrective eye movements during two-segment aiming movements. Moreover, older adults can maintain focus on a target for a longer duration than young adults. Interestingly, saccadic eye movements differ between people with glaucoma and those with normal vision, and can negatively impact the success of visually searching for target objects [21]. On the other hand, older people exhibit a significant increase in the deceleration phase of hand movements compared to young people [51]. This may be caused by sarcopenia since the natural aging process results in a loss of skeletal muscle mass and functionality [52]. Altered eye and hand movements may be adopted to increase the spatial extent of attention during task performance as a potential compensatory strategy for people with VF impairment.

A number of factors may help explain our observations. Several studies have investigated the relationship between glaucoma and cognitive impairment. Daveckaite et al. [17] found that normal-tension glaucoma patients had lower cognitive function scores and performed worse in a specific drawing style more often than those with cataracts. Bulut et al. [18] observed reduced cognitive performance (based on the Mini-Mental State Examination) in primary open-angle glaucoma patients and normal-tension glaucoma patients compared to the healthy group. Yochim et al. [53] diagnosed impairments in executive function in 22% of 41 glaucoma patients and memory impairment was found in 20% of those patients. Additionally, some evidence suggests glaucoma can be caused physical inactivity in daily life [8] which can accelerate age-related involutional changes in visuomotor processing.

This study is not without limitations, several of which are important to note. First, the cross-sectional study design precludes the establishment of a clear causal relationship between the long-term effects of glaucoma and visuomotor function. Moreover, samples used to test eye–hand coordination were obtained from patients at different glaucoma severities (moderate to advanced), and a wide range of ages, which may certainly limit inference. Further studies assessing a more homogeneous sample of patients with glaucoma may yield greater information as to how eye–hand coordination parameters are affected at different stages of the disease. Second, to increase inference, it may be important to confirm the significance of asymmetrical VF defects on eye–hand coordination efficiency by measuring other ophthalmological factors of visual disability such as asymmetrical structural damage, stereoacuity, and abnormal fixational eye movements, which should be considered in future studies.

## 5. Conclusions

Our findings demonstrate impaired eye–hand coordination in patients with moderate to advanced stages of glaucoma that are not observed in age-matched normal-sighted control subjects. Eye–hand coordination declines with advancing age and reduced visual acuity in glaucoma patients. Based on our data, monocular and binocular VF defects cannot fully explain the decline in eye–hand coordination in patients with glaucoma. Asymmetrical monocular VF defects are associated with poorer eye–hand coordination scores. We hypothesize that biological changes in the visuomotor system and interference of acceleration in patients’ age-related involutional changes of visuomotor processing may have contributed to these results.

## Figures and Tables

**Table 1:** Demographic and clinical characteristics of study participants.

Parameters	Glaucoma Patients, *n* = 28 Mean ± SD (Range/*n* [%])	Controls, *n* = 28 Mean ± SD (Range/*n* [%])	*p*
Age (year)	66.82 ± 6.52 (51–76)	65.54 ± 5.12 (51–73)	0.212
Women (%)	13 (46.43%)	14 (50%)	0.789
Left-handed (%)	1 (3.57%)	0 (0%)	0.313
Snellen BCVA			
Better eye (dB)	0.853 ± 0.147 (0.7–1.0)	1.021 ± 0.078 (0.9–1.2)	<0.001
Worse eye (dB)	0.671 ± 0.225 (0.1–1.0)	0.982 ± 0.061 (0.9–1.1)	<0.001
Monocular VF			
MD better eye (dB)	−5.848 ± 5.145 (−20.5–1.3)	0.720 ± 0.948 (−1.42–2.18)	<0.001
MD worse eye (dB)	−16.944 ± 8.224 (−32.4–6.4)	0.292 ± 0.941 (−1.45–1.78	<0.001
Binocular VF			
Defect scores (n)	25.821 ± 18.387 (0–75)	0.464 ± 0.999 (0–4)	<0.001
Esterman coefficient score (%)	78.482 ± 15.322 (37.5–100)	99.613 ± 0.833 (96.6–100)	<0.001

SD—standard deviation, BCVA—best corrected visual acuity, MD—mean deviation, VF—visual field.

**Table 2:** Mean, standard deviation (SD), median, and 25th and 75th percentile for each eye–hand coordination parameter in glaucoma patients (G) and control subjects (C), and intergroup comparisons using the Mann–Whitney U-test.

Parameters	Group	Mean ± SD	25th	Median	75th	*p*	Effect Size
**Aiming**							
Number of hits (n)	G	19.214 ± 1.134	19.000	19.000	20.000	0.491	-
	C	19.643 ± 0.621	19.000	20.000	20.000		
Total time (s)	G	9.801 ± 2.052	8.470	9.655	11.020	<0.001	0.46
	C	7.982 ± 1.596	7.170	7.635	8.735		
Aiming index (s^−1^)	G	2.029 ± 0.356	1.783	2.007	2.286	<0.001	0.47
	C	2.550 ± 0.490	2.172	2.542	2.721		
**Linear tracking**							
Number of errors (n)	G	31.610 ± 11.100	24.500	29.000	37.500	0.020	0.31
	C	24.286 ± 8.059	18.000	23.500	29.500		
Time of error (s)	G	2.929 ± 1.438	2.110	2.660	3.630	0.006	0.37
	C	2.046 ± 0.812	1.485	1.990	2.525		
Total time (s)	G	25.574 ± 11.006	18.110	22.025	33.945	0.724	-
	C	25.329 ± 12.651	15.170	20.855	32.170		
Tracking index (s^−1^)	G	0.168 ± 0.099	0.088	0.160	0.205	0.035	0.28
	C	0.214 ± 0.091	0.158	0.199	0.278		
**Tremor**							
Number of errors (n)	G	3.964 ± 7.280	0.000	3.000	5.000	0.011	0.34
	C	1.107 ± 2.671	0.000	0.000	1.000		
Time of error (s)	G	0.219 ± 0.286	0.000	0.095	0.325	0.014	0.33
	C	0.066 ± 0.205	0.000	0.000	0.045		
**Tapping**							
Number of hits (n)	G	183.036 ± 19.416	169.500	186.000	198.500	0.003	0.40
	C	199.929 ± 14.934	187.000	199.500	209.500		

**Table 3:** Spearman’s correlation coefficient for eye–hand coordination parameters, age, visual acuity, and VF defect severity (deviation scores for each eye, binocular VF score) in glaucoma patients.

Parameters	Age	BCVA Better Eye	BCVA Worse Eye	MD Better Eye	MD Worse Eye	MD Asymmetry	VF Binocular
**Aiming**							
Number of hits (n)	0.111	−0.151	−0.077	0.043	0.065	−0.184	0.072
Total time (s)	0.357	−0.467 *	0.029	−0.017	−0.290	0.369	0.120
Aiming index (s^−1^)	−0.374 *	0.507 *	−0.055	0.084	−0.155	−0.337	−0.092
**Linear tracking**							
Number of errors (n)	0.171	−0.297	−0.366	−0.081	0.170	−0.167	0.219
Error duration (s)	0.151	−0.300	−0.432*	−0.126	−0.441 *	0.405 *	−0.212
Total time (s)	−0.021	−0.023	0.028	−0.112	0.112	−0.230	0.125
Tracking index (s^−1^)	−0.062	0.149	0.165	0.122	−0.184	0.197	−0.195
**Tremor**							
Number of errors (n)	0.419 *	−0.444 *	0.003	−0.181	0.101	−0.017	−0.001
Error duration (s)	0.391 *	−0.465 *	−0.016	−0.232	0.075	−0.056	0.015
**Tapping**							
Number of hits (n)	−0.172	0.302	0.107	0.176	−0.085	0.254	0.120

* *p* < 0.05, BCVA—best corrected visual acuity, MD—mean deviation, VF—visual field.
